# A Phase I, first-in-human, open label, dose escalation study of MGD007, a humanized gpA33 × CD3 dual-affinity re-targeting (DART^®^) protein in patients with relapsed/refractory metastatic colorectal carcinoma

**DOI:** 10.1186/2051-1426-2-S3-P86

**Published:** 2014-11-06

**Authors:** Herbert Hurwitz, Todd Crocenzi, Joanna Lohr, Ezio Bonvini, Syd Johnson, Paul Moore, Jon Wigginton

**Affiliations:** 1 Duke University Medical Center, Durham, NC, Durham, United States; 2 Earle A. Chiles Research Institute, Portland, OR, Portland, United States; 3 MacroGenics, Inc., Rockville, MD, Rockville, United States

## 

Colorectal carcinoma (CRC) is the third-most commonly diagnosed cancer in the U.S., and the third-most common cause of cancer death. There were 136,830 new cases of CRC and 50,310 deaths due to CRC in 2014. For patients with unresectable metastatic disease, initial therapy commonly consists of chemotherapy combinations of Fluoropyrimidine agents, Oxaliplatin and Irinotecan with or without Bevacizumab or Cetuximab. Nonetheless, the prognosis of patients with metastatic CRC remains extremely poor overall, with 5-year survival rates estimated to be as low as 6-8%. There remains an urgent need, therefore, for new approaches for the treatment of patients with CRC.

Glycoprotein A33 (gpA33) is a 43 kDa membrane-bound glycoprotein with expression restricted to the surface of normal human colon and small bowel epithelial cells. In addition, the gpA33 antigen is homogeneously expressed at high levels in > 95% of primary and metastatic human CRC. These observations suggest that gpA33 that could be an attractive target for the immunotherapy of CRC. The Dual-Affinity Re-Targeting (DART^®^) technology, is a proprietary antibody-based platform that has been designed to engage multiple targets with a single molecule. MGD007 is a novel gpA33x CD3 DART protein that targets gpA33-positive cells for recognition and elimination by co-engagement of CD3-expressing T lymphocytes. MGD007 mediates redirected T cell killing of gpA33-expressing CRC cell lines in vitro and regression of transplantable CRC cell line xenografts in preclinical tumor models. Preclinical toxicology studies in cynomolgus monkeys demonstrated that MGD007 could be administered safely with a pharmacokinetic profile supportive of repeat dosing on a weekly basis.

We have designed a two-arm, Phase I dose-escalation study of MDG007 administered intravenously in patients with metastatic relapsed/refractory CRC. The study will employ a 3+3+3 design to explore the safety, PK, immunoregulatory activity and preliminary anti-tumor activity of MGD007 administered on either once-weekly or every three week schedules of administration. Projected doses to be tested will range from 0.6-150 mcg/kg. This study represents the first clinical evaluation of DART molecules in patients with solid tumors.

